# Antibiotic prophylaxis for caesarean section at a Ugandan hospital: a randomised clinical trial evaluating the effect of administration time on the incidence of postoperative infections

**DOI:** 10.1186/s12884-015-0514-3

**Published:** 2015-04-12

**Authors:** Lomangisi D Dlamini, Musa Sekikubo, Janat Tumukunde, Charles Kojjo, Davidson Ocen, Agnes Wabule, Arthur Kwizera

**Affiliations:** Department of Anaesthesia, Makerere University, P.O. Box 7072, Kampala, Uganda; Department of Obstetrics and Gynaecology, Makerere University, Kampala, Uganda

**Keywords:** Antibiotic prophylaxis, Caesarean section, Infection, Low-income setting

## Abstract

**Background:**

Prophylactic antibiotics are used to prevent postoperative infections after caesarean section. Studies have suggested that the timing of prophylaxis plays an important role. Over the years, the role of the anaesthesiologist in the administration of prophylactic antibiotics has become prominent. Therefore, there is an increasing need for anaesthesia providers to understand the rationale of antibiotic prophylaxis. We therefore sought to compare the effect of antibiotics prophylaxis within 1 hour before skin incision and after skin incision on the incidence of postoperative infections in patients undergoing caesarean section at Mulago Hospital.

**Methods:**

We conducted a single-blind randomised clinical trial conducted at Mulago Hospital evaluating 464 patients undergoing emergency caesarean section. Patients were randomly assigned a group number that allocated them to either arm of the study. They received the same prophylactic antibiotic according to their allotment, that is, either within 1 hour before skin incision or after skin incision as per current standards of practice in Mulago Hospital. They were followed up to detect infection up to 10 days postoperatively. The primary outcome was postoperative infection. The data collected were analysed with STATA version 12 using univariate and bivariate analysis.

**Results:**

The risk of overall postoperative infection was significantly lower when prophylaxis was given within an hour before incision (RR O.77, 95% CI 0.62–0.97). We also found endometritis to be significantly reduced in the pre-incision group (RR 0.62; 95% CI 0.39–0.99; P value 0.036).

**Conclusions:**

Giving prophylactic antibiotics before skin incision reduces risk of postoperative infection, in particular of endometritis.

**Trial registration:**

Pan African Clinical Trial Registry PACTR201311000610495.

Date of trial registration: 12^th^ August 2013.

## Background

A 2006 WHO systematic review of the causes of maternal death worldwide estimated that 9.7% of maternal deaths in Africa were due to puerperal sepsis [1]. One of the most important risk factors of postpartum infection in both developed and developing countries is caesarean section [2]. The second most common cause of maternal mortality is infection, and it contributes to 15% of the causes of maternal mortality in Uganda [3]. In low-income settings, improving the safety and care provided when performing a caesarean section can improve maternal and neonatal outcomes, which is in line with Millennium Development Goals number 4 and 5 [4].

Caesarean section is one of the most common surgical procedures performed in medical practice worldwide, and it is the most common surgical procedure performed in obstetrics and gynaecology practice at Mulago hospital. On average Caesarean section account for about 24% of 35 000 births per annum managed by Mulago hospital. Women giving birth by caesarean section present a 5- to 20-fold greater risk of infection than women giving birth by vaginal delivery [5]. Rates of severe wound infection can be as high as 25% [6]. One of the measures applied to prevent infectious complications following caesarean section is the use of prophylactic antibiotics [7,8]. The purpose of antibiotic prophylaxis in surgical procedures is not to sterilise tissues but to reduce colonisation pressure of microorganisms introduced at the time of the operation to a level that can be overcome by the patient’s immune system [9,10].

Whether antibiotic prophylaxis for caesarean section should be administered prior to the skin incision or at the time of the umbilical cord clamping remains controversial [11]. Traditionally, prophylaxis has been delayed in an effort to avoid masking a neonatal infection and to prevent an unnecessary septic workup. However, recent evidence shows no increase in neonatal sepsis, sepsis investigations, or length of stay [12-14]. A meta-analysis by Constantine et al. published in 2008 supports the use of antibiotic prophylaxis prior to caesarean incision to reduce total infectious morbidity without negative effects on neonatal outcomes [14]. Though more recent studies suggest there may be long-term effects of antibiotic prophylaxis on neonates, these still need to be validated by long-term follow-up studies of neonates exposed to antibiotics [15,16]. Thus, the American Congress of Obstetricians and Gynaecologists announced, in 2010, a new recommendation for antibiotic prophylaxis during caesarean delivery [17]. The new recommendation states that women giving birth by caesarean section should receive antibiotics routinely within 1 hour before the start of surgery. In the case of an emergency caesarean delivery, prophylaxis should be started as soon as possible. Further, a survey conducted in 2011 in the United States showed that 85% percent of patients received antibiotics preoperatively [18], indicating a change in the current practices in the country owing to the new recommendation. Very little is known about this practice in low-income countries in Africa, and in Uganda in particular.

At Mulago Hospital, there is evidence of increased postoperative infection in post-caesarean section patients as per an unpublished dissertation by Ssebuko. Currently there is no clear indication as to when antibiotic prophylaxis was administered and whether it contributed to the results of postoperative infection. Therefore, we sought to compare the relationship of timing of the administration of prophylactic antibiotics, within 1 hour before skin incision or after skin incision, and determine if either practice would affect the incidence of infection post-caesarean section.

## Methods

### Study design and setting

We conducted a single-blind randomised clinical trial, which was registered in the Pan African Clinical Trial Registry (Reference number PACTR201311000610495). We received ethical approval from our International Review Board called School of Medicine Research and Ethics Committee, and the study was conducted at Mulago National Referral Hospital in Kampala, Uganda, which is situated in Kampala, 2 km from the city centre. It is also the Makerere University Medical School teaching hospital, and it serves as a national referral hospital, as well as a general hospital for the people of Kampala and its surroundings. The obstetric and gynaecological wards are located in the 5^th^ floor, and they have two operating theatres: one for emergency cases from the labour ward and the other for elective cases, the Gynaecological theatre.

The sample size calculation was based on a similar study done in South Carolina, with an effect size of 40% [9]. For our study to have an 80% power and level of significance of 0.05, 421 patients were required. We added 10% to that value in case some were some patients were lost to follow-up, resulting in a total sample size of 464 patients.

### Eligibility

We included patients from the labour ward who had been scheduled for emergency caesarean section and provided written informed consent. Patients with evidence of current infection, that is, with fever, foul smelling discharge, already taking antibiotics, and those with allergic reaction to cephalosporins were excluded.

Upon providing informed consent, patients were allocated intervention according the number picked from randomisation envelopes. The patients were given consent forms that had an information sheet about the study and it was verbal assured that they understood and then signed if in agreement. We created four blocks of 116 patients with computer-generated numbers. Each number was accompanied by an A or B to specify which group a patient belonged to. Each block was kept in different sealed envelopes. Patients in group A were administered ceftriaxone 2 g intravenously 15 to 60 minutes before the skin incision, and those in group B were administered the same dose after performing the skin incision. The patients were blinded to the timing of the treatment received, but the investigator was not blinded as the timing of antibiotic administration had to be known. However, the investigators conducting the follow-up were blinded.

Patients were followed up in the postnatal ward on each of the 3 postoperative days to assess for signs and symptoms of infection, and their temperature was taken using digital thermometers. Infection was defined as endometritis (uterine tenderness, maternal tachycardia, fever), wound infection (surgical site infection according to criteria by the American Centre for Disease Control) [19] and fever (temperature greater than 37.8°C after 24 hours postoperatively). The last assessment for infection was performed on the 10th postoperative day as this was the date for patient’s postnatal check-up.

### Outcomes

The primary outcome was either the presence or absence of infection (either endometritis, fever morbidity or wound infection) during the observation period, which lasted until the 10th postoperative day. Secondary outcomes were neonatal outcomes reported during the time of observation, including any admission to the neonatal intensive care unit and treatment for infection. Infection was treated as per protocols from the Department of Obstetrics and Gynaecology.

### Data collection

Data were collected using interviewer-administered pre-tested questionnaires. Filled in questionnaires were checked daily for completeness and accuracy by the principal investigator, and errors were corrected before data entry on Epidata 3.1 (The EpiData Association, Odense, Denmark).

### Quality control

Research assistants were blinded as to which group patients were in. They were trained on how to assess and ask questions regarding symptoms, and we ensured that they were conversant with taking vital signs.

### Data analysis

Data were exported and analysed using STATA software version 12 (StataCorp Inc., Texas, USA). We conducted an interim analysis after half of the sample size was recruited.

Univariate analysis was used for basic characteristics of participants expressed as categorical and continuous variables, including age. Continuous variables were expressed as means and standard deviations, while categorical data were expressed as frequencies with their respective percentages.

The risk of postoperative infection in the two groups was assessed by taking the number of new infections as the numerator and the denominator was the total number of participants in each group. Binomial regression was used to estimate the risk ratio, with respective 95% confidence intervals, by taking the risk of infection among those who received antibiotics preoperatively as the numerator and postoperation risk as the denominator. The same methods were used to estimate the risk ratio of neonatal outcomes between the two groups. In all analyses, a p-value of < 0.05 was considered statistically significant.

### Adverse event monitoring

A Data Safety Management Board, comprising an anaesthesiologist, obstetrician, paediatrician and pharmacist, was to be notified of adverse events. Adverse events were defined as the presence of adverse neonatal outcomes in more than 15% of study patients. If this occurred, the study would be stopped and events treated as per protocol for management of neonatal sepsis in the Department of Paediatrics. At interim analysis, there were no reasons to stop the study because there were no reports of adverse neonatal outcomes. The study was continued until the sample size was achieved.

## Results

**Figure 1:**
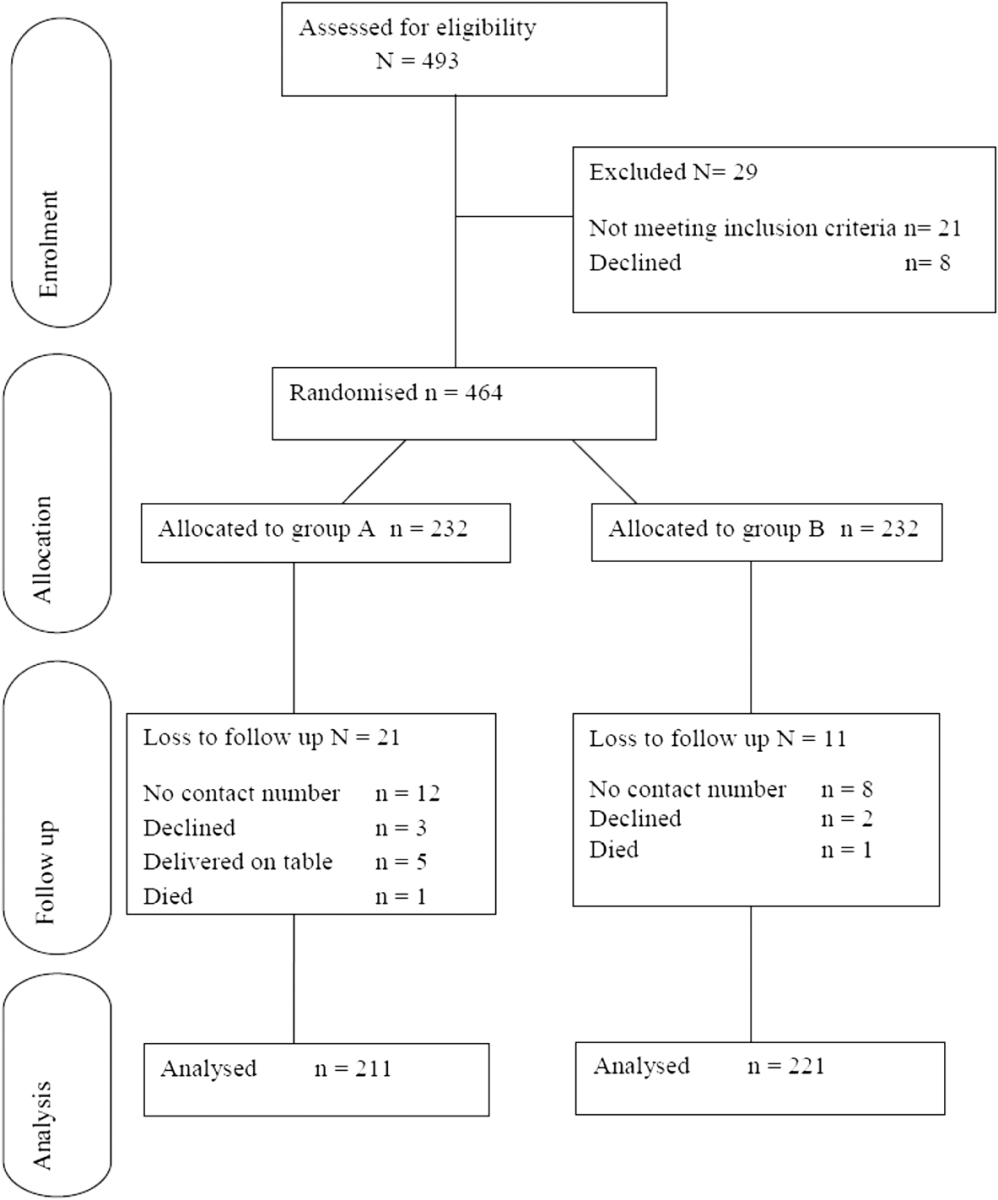
Patient flow diagram.

In this study, we assessed 493 patients who were scheduled for caesarean section between January 2014 and March 2014. Twenty-nine patients were excluded from the study: 21 did not meet the inclusion criteria and eight refused participation in the study. The remaining 464 patients were then randomised and received intervention as per allocated groups A (Pre-incision) or B (Post-incision). Of these, only 432 were analysed as the other 32 were lost to follow-up (Figure 1). This difference did not affect the power of the study because the sample size calculation indicated that 421 patients were needed for a power of 80% and level of significance of 0.05.

The distribution and characteristics of patients are shown in Table 1. The general characteristics were generally evenly distributed, except for type of incision and type of anaesthesia given, which were significantly different (P values < 0.05, respectively). These variables were considered later in a multivariate model to determine their effects on study outcomes (Table 2).

**Table 1:** **Distribution and general characteristics of patients in both groups**

**Variable**	**Randomisation group**		**Overall N (%)**	**P value**
	**Pre-incision**	**N (%)Post-incision**		
	**N (%)**	**N (%)**		
**Age group in years**				
**18**–**25**	124 (56.11)	121 (57.35)	245 (56.71)	
**26**–**35**	88 (39.82)	81 (38.39)	169 (39.12)	
**>35**	9 (4.02)	9 (4.27)	18 (4.17)	0.953
**Diabetes**				
**No**	210 (99.53)	218 (98.64)	428 (99.07)	
**Yes**	1 (0.47)	3 (1.36)	4 (0.93)	0.338
**HIV status**				
**Not known**	26 (12.32)	29 (13.12)	55 (12.73)	
**Positive**	23 (10.90)	25 (11.31)	48 (11.11)	
**Negative**	162 (76.78)	165 (75.57)	329 (76.16)	0.955
**Chronic medication**				
**No**	190 (90.05)	196 (88.69)	386 (89.35)	
**Yes**	21 (9.95)	25 (11.31)	46 (10.65)	0.647
**Previous Operation**				
**No**	107 (50.71)	126 (57.01)	233 (53.94)	
**Yes**	104 (49.29)	95 (42.99)	199 (46.06)	0.189
**Type of incision**				
**Midline**	106 (50.24)	86 (38.91)	192 (44.44)	
**Pfannenstiel**	105 (49.76)	135 (61.09)	240 (55.56)	0.018
**Anaesthesia given**				
**General**	32 (15.17)	62 (28.05)	94 (21.76)	
**Spinal**	179 (84.83)	159 (71.95)	338 (78.24)	0.001
**Intraoperative complications**				
**No**	200 (94.79)	206 (93.21)	406 (93.98)	
**Yes**	11 (5.21)	15 (6.79)	26 (6.02)	0.492

N, number; HIV, human immunodeficiency virus.

**Table 2:** **Measure of association of incidence of infection with significant general characteristics**

**Variable**	**Measure of association**		
	**Crude RR (95% CI)**	Adjusted RR (95% CI)*	**P value**
**Overall infections**	0.77 (0.62–0.97)	0.78 (0.62–0.97)	0.029
**Pfannenstiel incision**	0.90 (0.72–1.12)	0.93 (0.74–1.15)	0.503
**General anaesthesia**	0.98 (0.75–1.26)	1.01 (0.78–1.32)	0.918

*Adjusted for Overall infections, Pfannenstiel incision and general anaesthesia.

RR, risk ratio; CI, confidence interval.

On average, patients in group A received prophylaxis [mean (standard deviation)] 26.09 (9.67) minutes before the skin incision was performed. This timing falls within the 15 to 60 minutes that was specified in the protocol for this group. On average, patients in group B received prophylaxis 13.00 (12.93) minutes after the skin incision (Table 3).

**Table 3:** **Mean time between incision and prophylaxis and average length of operation in both groups**

**Variable**	**Overall**	**Pre-incision**	**Post-incision**	**Mean difference**	**P value**
**Time difference between prophylaxis and incision (minutes)**	19.40 (13.18)	26.09 (9.67)	13.00 (12.93)	13.09	0.001
**Length of operation (minutes)**	60.71 (120.7)	56.49 (168.6)	64.74 (36.9)	8.25	0.478

The data are presented as mean (standard deviation).

The primary outcomes are presented in Table 4. Overall, infection was observed in 65.9% (139/211) of patients in group A and in 85.1% (188/221) of patients in group B, and the difference between groups was statistically significant (risk ratio [RR] 0.77; 95% confidence interval [CI] 0.62–0.97; P value = 0.022). Wound infection was observed in 51.2% (108/211) in group A and 61.5% (136/221) in group B. Endometritis was seen in 14.7% (31/211) in group A and 23.5% (52/221) in group B, and the difference in the risk of endometritis was statistically significant (RR 0.62;95% CI 0.39–0.99; P = 0.036). Neonatal outcomes were seen in 1.4% (3/211) in group A and 0.4% (1/221) in group B, but the difference between groups was not statistically significant. These were neonates admitted into the neonatal intensive care unit during the observation period and were treated for infection.

**Table 4:** **Association between infection and time of antibiotic administration**

**Outcomes**	**Randomisation groups**				**Risk ratio (95% CI)**	**P value**
	**Pre-incision**		**Post-incision**			
	**Total population = 211**		**Total population = 221**			
	**Events**	**Risk**	**Events**	**Risk**		
**Postoperative infections**						
**Overall infections**	139	0.659	188	0.851	0.77 (0.62–0.97)	0.022
**Wound infection**	108	0.512	136	0.615	0.83 (0.64–1.08)	0.153
**Endometritis**	31	0.147	52	0.235	0.62 (0.39–0.99)	0.036
Neonatal outcomes*	3	0.014	1	0.004	3.14 (0.25–164.95)	0.352

*Admission to the Neonatal Intensive Care Unit and treatment for infection;

CI, confidence interval.

A multivariate model was used to assess the general characteristics of patients that were not evenly distributed between the groups, but that were significantly different. These included type of incision (P = 0.018) and type of anaesthesia (P = 0.001). As presented in Table 2, the risk of overall infection remained significantly lower in group A compared with group B, even after controlling for effects of Pfannenstiel incision and general anaesthesia.

## Discussion

We conducted a single-blind randomised clinical trial to compare the effect of the time of administration of prophylactic antibiotics on the risk of postoperative infection.

### Primary outcome

We found that the incidence of overall infection in group A (pre-incision) was lower than in group B (post-incision). In this study, we considered wound infection, endometritis and fever, occurring within an observation period of 10 days post-caesarean section as postoperative infections. Our results show that there was a statistically significant difference in this risk ratio, with less infection in the pre-incision group. Further, endometritis was significantly less frequent in the pre-incision group, whereas the between-group difference for wound infection was not statistically significant. These results are in accordance with the results of previous studies conducted in the United States [14,18,20]. None of the patients developed fever. It is possible that fever could have been truly absent or that its presence was masked by the pain control medication administered to all the patients, which were also antipyretic agents.

We found that risk of postoperative infection was higher in comparison with similar studies performed in different settings in developed countries [14,18,20]. Thus, we consider that greater efforts are still needed in aims to reduce the risk of post-caesarean infections. Our study only focused on one aspect of preventative measures—that is, the use prophylactic antibiotics. However, other measures, for example, adherence to aseptic procedures and quality of disinfectants need to be explored in this setting as well.

### Secondary outcome

The proportions of adverse neonatal outcomes were 1.4% and 0.4% in the pre-incision and post-incision groups, respectively. These were neonates admitted into the neonatal intensive care unit during the observation period and were treated for infection. One of the neonates that presented infection in the pre-incision group was admitted into the neonatal intensive care unit for intra-partum asphyxia. However, the outcome was not statistically significant, indicating that the timing of prophylaxis did not affect neonatal outcomes significantly. The entire neonatal period cannot be accounted for because the follow-up period in this study consisted of only 10 postoperative days. Thus, it is unknown if there were any long-term adverse effects, and this aspect needs to be explored further.

The diagnosis of infection in this study was based on clinical signs and symptoms. However, the quality of the study could have been improved if laboratory investigations were used. This was not planned as part of the protocol because the study was designed as a pragmatic clinical trial that mirrored local resources. When the patients were discharged to be followed up on day 10, we had no control of their environment and whether they took other medication. We consider this another limitation of the study because this factor may have affected the outcome, and this aspect was not explored in this study. Additionally, although some patients were lost to follow-up, the power of study was not affected because the number of patients lost was lower than that estimated by the sample size calculation. We, however, admit that another serious limitation to this study was its single-blind design.

## Conclusions

Administration of prophylactic antibiotics within 1 hour before skin incision significantly reduced the overall incidence of postoperative infections. Regarding the different types of infection, endometritis was significantly reduced. There were no significant adverse neonatal outcomes associated with the timing of antibiotic prophylaxis.

Based on the results of this study, we can recommend that prophylactic antibiotics be administered within 1 hour before skin incision in patients scheduled to undergo caesarean section in order to reduce the overall incidence of postoperative infection at Mulago Hospital. Additionally, we recommend the conduct of further investigations to determine other means of prevention of postoperative infections in an effort to reduce the incidence rate.

## References

[1] WHO (2008). Education Material for Teachers of Midwifery: Midwifery Education Modules - Second Edition, In Managing Puerperal Sepsis.

[2] Declercq E, Barger M, Cabral HJ, Evana SR, Kotelchuck M, Simon C (2007). Martenal outcomes associated with planned primary cesarean births compared with planned vaginal births. Obstet Gynecol.

[3] Freddie Ssengooba SN, Anthony Mbonye, Olive Sentubwe, Virgil Onama. Maternal Health Review Uganda, H.S. Development, Editor. 2003, Makerere University Institute of Public Health, Kampala, Uganda.

[4] United Nations Millennium Development Goals and Beyond 2015. www.un.org/millenniumgoals/

[5] Smaill FM, Gyte GM (2010). Antibiotic prophylaxis versus no prophylaxis for preventing infection after cesarean section. Cochrane Database Syst Rev.

[6] Henderson E, Love EJ (1995). Incidence of hospital-acquired infections associated with caesarean section. J Hosp Infect.

[7] J Shetty, S Rajshekhar, A Kamath. Short term antibiotic prophylaxis for emergency cesarean delivery: Is there a difference? The Internet Journal of Gynecology and Obstetrics. 2009;11(1).

[8] Alfirevic Z, Gyte GM, Dou L (2010). Different classes of antibiotics given to women routinely for preventing infection at caesarean section. Cochrane Database Syst Rev.

[9] Kaimal AJ, Zlatnik MG, Cheng YW, Thiet MP, Connatty E, Creedy P (2008). Effect of change in policy regarding the timing of prophylactic antibiotics on the rate of post caesarean delivery surgical-site infections. Am J Obstectrics Gynaecol.

[10] Mangram AJ, Horan TC, Pearson ML, Silver LC, Jarvis WR (1999). Guideline for prevention of surgical site infection, 1999. Hospital infection control practices advisory committee. Infect Control Hosp Epidemiol.

[11] Classen DC, Evans RS, Pestotnik SL, Horn SD, Menlove RL, Burke JP (1992). The timing of prophylactic administration of antibiotics and the risk of surgical-wound infection. N Engl J Med.

[12] Sullivan SA, Smith T, Chang E, Hulsey T, Vandorsten JP, Soper D (2007). Administration of cefazolin prior to skin incision is superior to cefazolin at cord clamping in preventing postcesarean infectious morbidity: a randomized, controlled trial. Am J Obstet Gynecol.

[13] Kafulafula G, Mwatha A, Chen YQ, Aboud S, Martinson F, Hoffman I (2009). Intrapartum antibiotic exposure and early neonatal, morbidity, and mortality in Africa. Pediatrics.

[14] Costantine MM, Rahman M, Ghulmiyah L, Byers BD, Longo M, Wen T (2008). Timing of perioperative antibiotics for cesarean delivery: a metaanalysis. Am J Obstet Gynecol.

[15] Lamont RF, Sobel JD, Kusanovic JP, Vaisbuch E, Mazaki-Tovi S, Kim SK (2011). Current debate on the use of antibiotic prophylaxis for caesarean section. BJOG.

[16] McKeever TM, Lewis SA, Smith C, Hubbard R (2002). The importance of prenatal exposures on the development of allergic disease. Am J Respir Crit Care Med.

[17] The American College of Obstetricians and Gynecologists, Women’s Health Care Physicians (2010). Committee opinion no. 465: antimicrobial prophylaxis for cesarean delivery: timing of administration. Obstet Gynecol.

[18] Baaqeel H, Baaqeel R (2013). Timing of administration of prophylactic antibiotics for caesarean section: a systematic review and meta-analysis. BJOG.

[19] Horan TC, Gaynes RP, Martone WJ, Jarvis WR, Emori TG (1992). CDC definitions of nosocomial surgical site infections, 1992: a modification of CDC definitions of surgical wound infections. Infect Control Hosp Epidemiol.

[20] Owens SM, Brozanski BS, Meyn LA, Wiesenfeld HC (2009). Antimicrobial prophylaxis for cesarean delivery before skin incision. Obstet Gynecol.

